# Magnitude and meaningfulness of change in SF-36 scores in four types of orthopedic surgery

**DOI:** 10.1186/1477-7525-6-55

**Published:** 2008-07-31

**Authors:** Lucy Busija, Richard H Osborne, Anna Nilsdotter, Rachelle Buchbinder, Ewa M Roos

**Affiliations:** 1 Centre for Rheumatic Diseases, Department of Medicine (Royal Melbourne Hospital), the University of Melbourne, Melbourne, Australia; 2 R&D Department, Halmstad Central Hospital, Halmstad, Sweden; 3 Monash Department of Clinical Epidemiology at Cabrini Hospital, Department of Epidemiology and Preventive Medicine, Monash University, Melbourne, Australia; 4 Department of Orthopedics, Clinical Sciences Lund, Lund University, Sweden; 5 Institute of Sports Science and Clinical Biomechanics, University of Southern Denmark, Odense, Denmark

## Abstract

**Background:**

The Medical Outcomes General Health Survey (SF-36) is a widely used health status measure; however, limited evidence is available for its performance in orthopedic settings. The aim of this study was to examine the magnitude and meaningfulness of change and sensitivity of SF-36 subscales following orthopedic surgery.

**Methods:**

Longitudinal data on outcomes of total hip replacement (THR, n = 255), total knee replacement (TKR, n = 103), arthroscopic partial meniscectomy (APM, n = 74) and anterior cruciate ligament reconstruction (ACL, n = 62) were used to estimate the effect sizes (ES, magnitude of change) and minimal detectable change (sensitivity) at the group and individual level. To provide context for interpreting the magnitude of changes in SF-36 scores, we also compared patients' scores with age and sex-matched population norms. The studies were conducted in Sweden. Follow-up was five years in THR and TKR studies, two years in ACL, and three months in APM.

**Results:**

On average, large effect sizes (ES≥0.80) were found after orthopedic surgery in SF-36 subscales measuring physical aspects (physical functioning, role physical, and bodily pain). Small (0.20–0.49) to moderate (0.50–0.79) effect sizes were found in subscales measuring mental and social aspects (role emotional, vitality, social functioning, and mental health). General health scores remained relatively unchanged during the follow-up. Despite improvements, post-surgery mean scores of patients were still below the age and sex matched population norms on physical subscales. Patients' scores on mental and social subscales approached population norms following the surgery. At the individual level, scores of a large proportion of patients were affected by floor or ceiling effects on several subscales and the sensitivity to individual change was very low.

**Conclusion:**

Large to moderate meaningful changes in group scores were observed in all SF-36 subscales except General Health across the intervention groups. Therefore, in orthopedic settings, the SF-36 can be used to show changes for groups in physical, mental, and social dimensions and in comparison with population norms. However, SF-36 subscales have low sensitivity to individual change and so we caution against using SF-36 to monitor the health status of individual patients undergoing orthopedic surgery.

## Background

The Medical Outcomes Study Short Form Health Survey (SF-36) is a health status questionnaire that was developed almost 20 years ago for the assessment of functional status and well-being [1]. Its 36 items assess eight health-related concepts thought to be affected by disease and treatment interventions: physical functioning, role limitations due to physical health problems (role physical), bodily pain, general health, energy levels/fatigue (vitality), social functioning, role limitations due to emotional problems (role emotional), and psychological distress (mental health). The SF-36 has been applied in a variety of clinical settings [2-6] including orthopedic surgery where it has been frequently used to evaluate psychometric and clinometric properties of other self-report questionnaires [7-9].

The popularity of the SF-36 is in part related to accumulating support for its satisfactory validity and reliability across study settings and populations [10-13]. Population norms for SF-36, by age and sex, are available for several countries, allowing comparisons of the health status of the patient groups with the general population [1,14-16]. To be of practical use in clinical and research settings, measures that are used to assess outcomes of an intervention must have been shown to be able to detect change in health status. Given that statistical significance of change is sample-dependent (in large studies minute and clinically unimportant changes may be statistically significant and fallaciously regarded as clinically significant), the magnitude of change (effect size) following an intervention is more informative to clinical practitioners. Information on effect size is also useful in research settings, where it can be used to calculate the sample size required to detect changes of a certain magnitude.

An additional measurement issue associated with comparing pre- and post-intervention scores is that change scores may be due to random measurement error, real change in health status, or both. Therefore, an important characteristic of a sound measure is the ability to detect meaningful change in participants' health state. The ability of a questionnaire to detect a meaningful change is known as sensitivity, with instruments that are more sensitive being able to detect smaller changes. Ideally, the measurement properties of a questionnaire should be tested in the settings in which it will be used. However, relatively few studies have specifically examined the magnitude and meaningfulness of changes in SF-36 scores following orthopedic surgery, and mixed results have been reported in those that have [9,17,18].

The aim of this study was to assess the utility of SF-36 subscales in orthopedics by examining the magnitude and meaningfulness of change and sensitivity of SF-36 scores in orthopedic surgery. To provide context for interpreting the magnitude of changes in SF-36 scores, we also compared patients' pre- and post-operative scores with the age and sex adjusted population norms.

## Methods

To estimate magnitude of change and sensitivity of SF-36 subscales in orthopedic settings, we utilized secondary data from prospective follow-up studies of outcomes in total hip replacement (THR), total knee replacement (TKR), arthroscopic partial meniscectomy (APM), and anterior cruciate ligament (ACL) reconstruction surgery. The methods of these studies have been previously published and are summarized here only briefly.

### Total hip replacement (THR) groups

This group included 274 consecutive patients having THR for hip osteoarthritis at the Department of Orthopedics at Halmstad Central Hospital, Sweden and 110 controls, matched to the patients by age, sex and municipality [19]. Controls were identified from the Swedish National Population Records. In all, 258 eligible controls were identified, with 45% (n = 116) agreeing to take part in the study. After exclusion of those who reported hip complaints (pain or diminished range of motion) (n = 6), the remaining number (110) was regarded as sufficient for group comparisons. Patients' mean age was 70.5 years and 53% were women. Mean age of controls was 70.7 years and 55% were women. Patients were assessed before the surgery (baseline) and reassessed at six months and five years after the surgery. Controls were assessed at the time of recruitment, with follow-up assessments also at six months and five years. Five-year follow-up rates were 65% for both groups (Table 1).

**Table 1:** Follow-up rates for the study groups

Group	Number of participants (% of baseline)					
	
	Baseline	3 months	6 months	1 year	2 years	5 years
Total hip replacement (controls)	110	-	74 (67%)	-	-	71 (65%)
Total hip replacement (patients)	274	-	222 (81%)	-	-	179 (65%)
Total knee replacement	105	-	94 (90%)	87 (83%)	-	80 (76%)
Arthroscopic partial meniscectomy	74	63 (85%)	-	-	-	-
Anterior cruciate ligament reconstruction	62	-	62 (100%)	55 (89%)	46 (74%)	-

#### Total knee replacement (TKR) group

This group included data from 105 consecutive patients having TKR for knee osteoarthritis at the Department of Orthopedics at Lund University Hospital, Sweden. Their mean age was 71.3 years and 63% were women [20]. Patients were assessed before the surgery (baseline), with follow-ups at six months, one year, and five years. At final follow-up data were available from 76% of patients.

#### Arthroscopic partial meniscectomy (APM) group

This group included 74 consecutive patients from Department of Orthopedics at Lund University Hospital, Sweden who received arthroscopic partial meniscectomy as the only intervention. Their mean age was 44.8 years and 32% were women [21]. The assessments were conducted before the surgery (baseline) and three months after the surgery (85% follow-up rate).

#### Anterior cruciate ligament (ACL) reconstruction group

This group included data from 62 Swedish patients randomized to an ACL reconstruction within a trial of surgical versus non-surgical treatment of acute ACL tear (ISRCTN 84752559). Inclusion criteria were age between 18 and 35 years, having a moderate to high physical activity level and no more than four weeks since ACL rupture at time of reconstruction. Their mean age was 25.9 years and 19% were women [22]. Patients were assessed before surgery (baseline), with follow-ups at six months, one year, and two years (74% follow-up rate).

### Ethical approval and informed consent

Research carried out for the studies reported here complies with the Helsinki Declaration. Each study was approved by the Ethics Committee of the Medical Faculty of Lund University, Lund, Sweden. Written informed consent was obtained from the participants for the publication of results. Copies of the written consent are available for review by the Editor-in-Chief of this journal.

### Measures

All study groups were administered SF-36 at each assessment. The SF-36 is a self-report generic health status questionnaire comprised of eight subscales: physical functioning (PF), role physical (RP), bodily pain (BP), general health (GH), vitality (VT), social functioning (SF), role emotional (RE), and mental health (MH) [23-25]. The scores range between 0 and 100, with higher scores representing better health.

### Statistical analyses

The original data for each study were extracted for the analyses.

#### Effect sizes

Magnitude of change in SF-36 subscale scores was assessed using Cohen's *d *[26]. Cohen's *d *is a standardized measure of effect size (ES) and provides information on the amount of change in the measure relative to the variation within the measure. Cohen's *d *is computed as the difference between the baseline and follow-up scores divided by the standard deviation of baseline scores. Benchmarks to classify the importance of the change are available, with ES values of 0.20–0.49 considered small, values of 0.50–0.79 considered moderate, and values ≥ 0.80 considered large [26]. ES were calculated so that positive values represent improvement and negative values represent deterioration.

Given that questionnaire change scores cannot be reliably estimated for the participants with extreme scores, we also examined the presence of floor and ceiling effects at each assessment time. The subscales were deemed to have floor or ceiling effects if 15% of respondents or more reported the worst (0) or best (100) possible scores, respectively.

#### Sensitivity

Sensitivity of subscales was evaluated using Minimal Detectable Change (MDC), calculated at individual and group levels. While individual and group MDC are related concepts, they convey different information. Individual level MDC provide information on whether observed changes in the individual's health status are greater than chance variations [27] whereas group level MDC are useful for comparing meaningfulness of change across samples [28].

Differences in the scores on the same measure obtained on different occasions may be due to random error, real change in health status, or a combination of both [27]. Therefore, MDC used for this study was based on standard error of measurement (SEM). Since the smaller the measurement error, the smaller the changes can be de detected beyond random error, with lower values of SEM indicating more sensitive subscales. SEM was derived from within subjects analysis of variance [29] with time of assessment (i.e., baseline, follow-up) as the within subjects factor [30]. This study design partitions the within-person variations in SF-36 scores into between-assessment variance and the residual variance [30]. The former represents systematic differences between assessment times, such as intervention effects, while the latter represents residual variance due to random error and error from unknown systematic sources. SEM was calculated as a square root of this residual within person variance [30]. To determine with 95% confidence whether observed changes were larger than the random error, individual level MDC (MDC_ind_) were calculated as 1.96*√2*SEM [29,31-33]. Group level MDC (MDC_grp_) were based on standard errors of the sample means. Standard error of the mean is influenced by both the within-subjects variability and the sample size, therefore MDC_grp _were calculated as (1.96*√ 2*SEM)/√n [32,34,35]. The differences in group scores between baseline and follow-ups were interpreted as 'real' change if they exceed values of MDC [28].

MDC reflects changes that are greater than measurement error (i.e., statistically significant change) and should not be equated with clinically important change (change that clinicians and patients regard as important). Since minimal clinically important changes (MCIC) for SF-36 subscales are not well studied in orthopedic settings, we utilized the published standards for minimal "clinically and socially relevant" change in group scores as a measure of MCIC at a group level [36]. The standards for clinically and socially relevant changes at a group level are based on Cohen's *d*, with minimal important change represented by a moderate effect size (0.50–0.79), which corresponds to at least 5-point change in scores on the 0–100 scale (5%) [36]. SF-36 subscales with MDC_grp _less than five were considered to have acceptable sensitivity to change in group scores. To determine whether the observed changes in SF-36 scores were statistically and clinically meaningful, we also compared the average group changes with values of MDC group and MCIC, respectively.

Established standards for MCIC at an individual level are essential for interpretation of intra-individual change as they help to determine clinical meaningfulness of the observed change in individual scores. Estimates of individual level MCIC are also important for evaluating sensitivity of a measure since a scale can only be regarded as sufficiently sensitive to detect meaningful changes in individual health status if the values of MDC_ind _do not exceed values of individual level MCIC [33,37]. However, generally accepted standards for individual level MCIC in orthopedic surgery currently do not exist. Since scale's sensitivity to change is affected by measurement error, we used values of 95% confidence intervals (CI; calculated as 1.96*SEM) around SF-36 scores from a normative population-based sample [36] to gauge measurement error in SF-36 scores in orthopedic settings. As the CI and MDC represent boundary for true score and boundary for change, respectively, change could not be regarded as 'real' if the amount of measurement error around the true score exceeded the amount of measurement error around the change score. Therefore, SF-36 subscales were regarded as sufficiently sensitive to detect real changes in individual scores if MDC_ind _were smaller than the normative values of 95% CI: 12 points for PF, 23 points for RP, 15 points for BP, 18 points for GH, 16 points for VT, 26 points for SF, 28 points for RE, and 24 points for MH subscales [36]. It is important to note however that CI values were used as an external standard for the expected amount of measurement error in SF-36 scores and not as a substitute for individual level MCIC.

#### Proportion improved or deteriorated

MDC_ind _was used to categorize change in participants' scores. Those who had scores that decreased by an amount greater than the MDC_ind _were classified as 'worse'; those whose scores increased by an amount greater than the values of MDC_ind _were classified as 'better', and those with change scores less than or equal to MDC_ind _were classified as 'no change'.

#### Population norm comparisons

To provide context for interpreting changes in health status following orthopedic surgery, patients' SF-36 scores were compared with the published norms for SF-36 for the Swedish population of the same age and sex [1,15]. As the standard errors for the published norm scores were very small, the mean values of the normative scores were used to represent the 'real' values for the population of each age and sex group. Average group scores within +/- 5 points of the population norm were considered to be within the norm [1,36].

All statistical analyses were performed using SPSS Version 15. Longitudinal changes were calculated using data from participants with complete follow-up only.

## Results

SF-36 baseline data were available for 515 patients who underwent orthopedic surgery, including 274 THR, 105 TKR, 74 APM, and 62 ACL reconstruction patients. In the THR study, there were also 110 age and sex matched controls. Follow-up rates for the patients varied between 81% (APM) and 100% (ACL) at first post-surgical assessment (three months in APM study and six months in THR, TKR, and ACL studies) and between 65% (THR) and 76% (TKR) at final follow-up (two years for the ACL and five years for THR and TKR studies), see Table 1. Demographic characteristics are in Table 2. The proportion of men varied from 37% in TKR study to 81% in ACL study. On average, patients in the ACL study were youngest (mean [sd] 25.9 [5.1] at baseline), while patients in TKR study were the oldest (71.3 [8.1] years at baseline).

**Table 2:** Age and sex characteristics of the study groups at baseline

Group	% male	Age	
		
		M (SD)	Range
Total hip replacement (controls)	44.6	70.7 (7.6)	52–86
Total hip replacement (patients)	47.2	70.5 (8.9)	41–96
Total knee replacement	37.1	71.3 (8.1)	43–86
Arthroscopic partial meniscectomy	67.6	44.8 (12.2)	14–75
Anterior cruciate ligament reconstruction	80.6	25.9 (5.1)	18–35

### Baseline Scores

**Figure 1:**
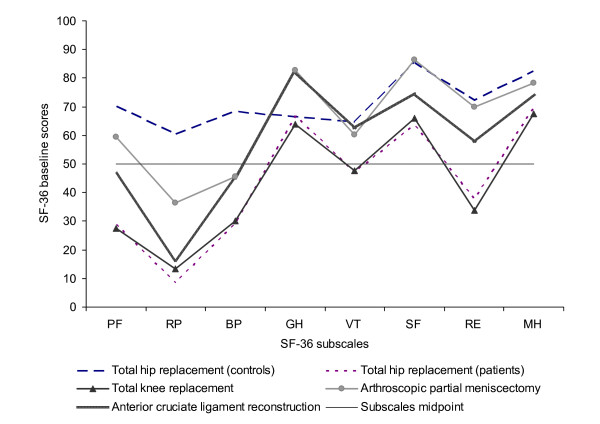
**Baseline SF-36 scores of the study groups**. Note: PF = Physical Functioning, RP = Role Physical, BP = Bodily Pain, GH = General Health, VT = Vitality, SF = Social Functioning, RE = Role Emotional, MH = Mental Health.

Average baseline scores are presented in Figure 1. The overall pattern of SF-36 subscale scores was similar across groups, with lowest scores recorded on RP subscale in all groups. The scores on GH, SF, and MH subscales tended to be similar within the groups and were generally better than the scores on other subscales. The greatest difference between the best and the worst subscale scores was observed for the ACL patients (GH versus RP subscales). THR and TKR patients had the worst baseline scores across all SF-36 subscales, and were well below the mid point (50 points) scores on PF, RP, BP, and SF subscales.

### Changes in SF-36 Scores

Average SF-36 scores of the study groups at baseline and at first and final follow-ups are presented in Table 3. While the THR control group did not change or deteriorated slightly, the intervention groups generally improved in their SF-36 scores during the follow-up. One exception was the GH subscale, with small deteriorations relative to baseline scores recorded for THR and TKR groups at five years and for the APM group at three months follow-up.

**Table 3:** Average SF-36 subscale scores and effect sizes for the study groups at first and final follow-up*

SF-36 scores		Total hip replacement (controls)			Total hip replacement (patients)			Total knee replacement			Arthroscopic partial meniscectomy			Anterior cruciate ligament reconstruction		
		
		N	M (SD)	ES	N	M (SD)	ES	N	M (SD)	ES	N	M (SD)	ES	N	M (SD)	ES
PF	Baseline	44	79.6 (17.7)		147	30.7 (20.1)		68	30.0 (14.9)		62	59.0 (21.8)		46	44.2 (21.8)	
	First follow-up	44	78.2 (21.8)	-0.1	147	60.5 (22.0)	1.5	68	60.6 (21.1)	2.1	62	73.7 (21.9)	0.7	46	79.6 (17.7)	1.6
	Final follow-up	44	74.5 (24.1)	-0.3	147	57.6 (27.3)	1.3	68	52.3 (24.1)	1.5				46	83.4 (20.2)	1.8
RP	Baseline	42	68.5 (41.0)		139	8.5 (20.2)		64	12.6 (23.7)		62	36.7 (38.3)		46	14.1 (26.7)	
	First follow-up	42	69.6 (42.6)	0.0	139	49 (42.6)	2.0	64	42.7 (42.4)	1.3	62	62.5 (42.2)	0.7	46	64.7 (40.0)	1.9
	Final follow-up	42	60.1 (42.4)	-0.2	139	49.6 (43.2)	2.0	64	48.0 (43.9)	1.5				46	80.4 (34.9)	2.5
BP	Baseline	50	75.7 (24.2)		154	30.9 (17.2)		66	30.6 (18.8)		62	44.4 (19.2)		46	41.8 (20.4)	
	First follow-up	50	73.0 (27.6)	-0.1	154	70.3 (23.6)	2.3	66	70.9 (23.7)	2.1	62	63.3 (24.9)	1.0	46	74.4 (20.7)	1.6
	Final follow-up	50	70.2 (28.0)	-0.2	154	67.1 (26.0)	2.1	66	63.9 (25.1)	1.8				46	75.8 (25.3)	1.7
GH	Baseline	46	70.2 (20.3)		139	68.8 (19.1)		59	66.0 (18.3)		61	82.4 (15.1)		46	81.5 (15.8)	
	First follow-up	46	68.6 (22.0)	-0.1	139	72.5 (20.7)	0.2	59	70.0 (20.9)	0.2	61	80.1 (19.4)	-0.2	46	85.0 (15.8)	0.2
	Final follow-up	46	61.8 (22.7)	-0.4	139	63.6 (22.9)	-0.3	59	62.7 (24.0)	-0.2				46	83.4 (17.1)	0.1
VT	Baseline	45	69.8 (21.7)		135	50.9 (20.1)		59	50.3 (26.7)		62	60.8 (22.1)		46	59.5 (19.3)	
	First follow-up	45	69.1 (21.6)	0.0	135	70.9 (19.2)	1.0	59	67.3 (24.4)	0.6	62	69.4 (22.3)	0.4	46	71.6 (22.5)	0.6
	Final follow-up	45	63.8 (22.6)	-0.3	135	64.3 (22.4)	0.7	59	61.0 (27.7)	0.4				46	72.1 (20.0)	0.7
SF	Baseline	49	87.8 (19.7)		157	65.4 (26.2)		66	72.7 (23.0)		62	86.3 (18.6)		46	72.6 (26.0)	
	First follow-up	49	84.9 (18.9)	-0.1	157	87.9 (19.5)	0.9	66	86.7 (19.1)	0.6	62	87.5 (22.6)	0.1	46	90.8 (16.1)	0.7
	Final follow-up	49	82.7 (24.0)	-0.3	157	84.3 (22.3)	0.7	66	83.5 (25.2)	0.5				46	94.3 (14.6)	0.8
RE	Baseline	37	76.1 (34.4)		139	39.3 (43.6)		56	40.5 (43.0)		62	68.8 (38.1)		46	52.9 (43.6)	
	First follow-up	37	76.6 (37.6)	0.0	139	68.1 (39.7)	0.7	56	64.0 (43.1)	0.5	62	77.4 (36.6)	0.2	46	81.9 (36.3)	0.7
	Final follow-up	37	77.5 (40.9)	0.0	139	65.5 (42.0)	0.6	56	57.7 (42.4)	0.4				46	92.0 (20.1)	0.9
MH	Baseline	45	86.6 (13.7)		136	69.8 (21.6)		59	71.0 (21.0)		62	78.1 (18.4)		46	71.8 (18.9)	
	First follow-up	45	85.2 (13.9)	-0.1	136	83.8 (17.7)	0.6	59	80.0 (19.7)	0.4	62	83.6 (17.6)	0.3	46	84.3 (17.0)	0.7
	Final follow-up	45	82.0 (15.6)	-0.3	136	80.6 (17.9)	0.5	59	77.2 (20.1)	0.3				46	86.2 (12.8)	0.8

*Note: First follow-up was three months for APM and six months for THR, TKR, and ACL groups; Final follow-up was five years for THR and TKR groups and two years for ACL.

PF = Physical Functioning, RP = Role Physical, BP = Bodily Pain, GH = General Health, VT = Vitality, SF = Social Functioning, RE = Role Emotional, MH = Mental Health.

#### Effect sizes

**Figure 2:**
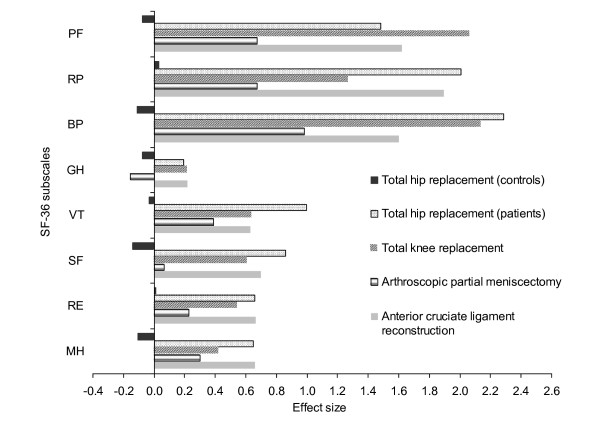
Effect sizes for SF-36 subscales across the study groups at first follow-up*. * Note: First follow-up was three months for APM and six months for TKR, THR, and ACL groups. PF = Physical Functioning, RP = Role Physical, BP = Bodily Pain, GH = General Health, VT = Vitality, SF = Social Functioning, RE = Role Emotional, MH = Mental Health.

ES for the first follow-up are presented in Figure 2 and in Table 3. Generally, the magnitude of changes in SF-36 was similar for patients in THR, TKR, and ACL groups, with smaller changes in the APM group. In the THR study, large improvements (ES≥0.80) at first follow-up occurred in PF, RP, BP, VT and SF scores, moderate improvements (ES 0.50–0.79) in RE and MH scores and small change in GH scores (ES = 0.20). For TKR patients, improvements at first follow-up were large in PF, RP, and BP scores, moderate in VT, SF, and RE scores and small in GH and MH scores. Improvements for APM patients could be classified as large on BP subscale only, with moderate improvements on PF and RP, small improvements on VT, RE, and MH, no change on SF, and a small deterioration on GH subscale. In the ACL study, improvements at first follow-up were large in PF, RP, and BP scores, moderate in VT, SF, RE, and MH scores, and small in GH scores.

The ES across SF-36 subscales have changed only slightly over time, with similar values recorded for fist and final follow-ups (see Table 3). In the studies where data were available on intermediate follow-up (one year after the surgery in TKR and the ACL groups) ES were generally highest at one year (data not shown).

#### Floor and ceiling effects

Baseline floor effects, indicating worst possible scores, were present in the RP subscale for all groups and the RE subscale for THR, TKR, and ACL groups (see Table 4). More troublesome for documenting potential improvements in scores were ceiling effects at baseline, which were observed in the SF and RE subscales for all groups and in the RP and GH subscales for APM group. Ceiling effects generally increased during the follow-up. PF and VT were the only subscales that displayed no ceiling effects at baseline or at follow-ups across all surgical groups.

**Table 4:** Floor and ceiling effects for SF-36 subscale scores for the study groups at first and final follow-up*

SF-36 scores		Total hip replacement (controls)		Total hip replacement (patients)		Total knee replacement		Arthroscopic partial meniscectomy		Anterior cruciate ligament reconstruction	
		
		% scoring 0	% scoring 100	% scoring 0	% scoring 100	% scoring 0	% scoring 100	% scoring 0	% scoring 100	% scoring 0	% scoring 100
PF	Baseline	-	12.0	8.3	-	1.4	-	1.6	0.0	2.2	-
	First follow-up	-	13.6	1.3	0.7	-	1.4	-	6.3	-	4.3
	Final follow-up	2.2	8.7	3.9	-	2.7	-			-	**28.3**
RP	Baseline	**19.6**	**54.3**	**80.8**	2.0	**70.4**	2.8	**39.7**	**19.0**	**76.1**	-
	First follow-up	**21.4**	**61.9**	**35.3**	**31.7**	**40.8**	**26.8**	**20.6**	**50.8**	**19.6**	**45.7**
	Final follow-up	**26.2**	**42.9**	**35.3**	**35.3**	**38.0**	**19.4**			10.9	**71.7**
BP	Baseline	-	**40.0**	9.0	1.3	8.3	1.4	-	1.6	-	2.2
	First follow-up	-	**36.0**	0.6	**27.1**	-	**26.4**	-	12.7	-	**21.7**
	Final follow-up	2.0	**38.0**	0.6	**25.8**	-	**19.4**			2.2	**41.3**
GH	Baseline	-	8.7	-	4.3	-	1.5	-	**16.1**	-	8.7
	First follow-up	-	13.0	0.7	5.7	-	8.8	-	**27.4**	-	13.0
	Final follow-up	-	6.5	1.4	4.3	-	8.8			-	**21.7**
VT	Baseline	-	4.4	2.2	0.7	3.0	4.5	-	3.2	-	-
	First follow-up	-	11.1	0.7	5.1	-	7.6	-	7.9	-	13.0
	Final follow-up	2.2	4.4	0.7	4.4	3.0	4.5			-	6.5
SF	Baseline	-	**64.0**	3.2	**17.7**	-	**22.5**	-	**52.4**	2.2	**32.6**
	First follow-up	-	**53.1**	0.6	**60.8**	-	**57.7**	-	**68.3**	-	**67.4**
	Final follow-up	2.0	**52.0**	1.9	**55.7**	2.8	**53.5**			-	**84.8**
RE	Baseline	10.8	**59.5**	**47.5**	**30.2**	**46.9**	**28.1**	14.3	**54.0**	**30.4**	**41.3**
	First follow-up	13.5	**67.6**	**17.3**	**55.4**	**28.1**	**53.1**	12.7	**68.3**	**15.2**	**76.1**
	Final follow-up	**18.9**	**75.7**	**23.0**	**54.0**	**26.6**	**43.8**			-	**84.8**
MH	Baseline	-	**20.0**	-	6.6	-	12.1	-	9.5	-	-
	First follow-up	-	**15.6**	0.7	**24.1**	-	**21.2**	-	**22.2**	-	**21.7**
	Final follow-up	-	13.3	-	**19.0**	-	**16.7**			-	**15.2**

*Note: First follow-up was three months for APM and six months for THR, TKR, and ACL groups; Final follow-up was five years for THR and TKR groups and two years for ACL.

PF = Physical Functioning, RP = Role Physical, BP = Bodily Pain, GH = General Health, VT = Vitality, SF = Social Functioning, RE = Role Emotional, MH = Mental Health.

Values indicating floor (15% or more with a score of 0) and ceiling (15% or more with a score of 100) effects are bolded.

#### Sensitivity: Group changes

The values of MDC_grp _varied across the study groups and across the subscales but were generally lager than or equal to the values of MCIC (5 points or more), see Table 5. This suggests that at least some of the meaningful changes in group scores could not be detected with 95% confidence. The observed changes in the average SF-36 subscale scores however were larger than either the values of MDC_grp _or MCIC across all intervention groups, indicating that statistically and clinically meaningful change in subscale scores had occurred following orthopedic surgery. Overall, GH subscale had the best ability to detect MCIC in orthopedic surgery, with MDC_grp _values of five or less in all intervention groups (Table 5). RP and RE subscales had the worst ability to detect MCIC in group scores, with values of MDC_grp _ranging from 8 (THR patients) to 12 (TKR and ACL) and from 9 (THR and APM) to 14 (TKR), respectively.

**Table 5:** Change in SF-36 subscales across study groups

	Norm 95%CI^¶^	Total hip replacement (controls)				Total hip replacement (patients)				Total knee replacement				Arthroscopic partial meniscectomy				Anterior cruciate ligament reconstruction			
		
		SEM*	MDC^#^		ΔM (SD)	SEM	MDC		ΔM (SD)	SEM	MDC		ΔM (SD)	SEM	MDC		ΔM (SD)	SEM	MDC		ΔM (SD)
																
			Ind	Grp			Ind	Grp			Ind	Grp			Ind	Grp			Ind	Grp	
PF	12	12	34	5	-2 (12)	18	49	4	27 (23)	15	41	6	29 (17)	16	45	6	15 (23)	14	40	6	34 (21)
RP	23	21	57	10	-2 (26)	33	91	8	33 (33)	30	84	12	35 (34)	32	88	11	27 (45)	29	81	12	50 (30)
BP	15	15	41	6	-3 (16)	20	54	5	37 (23)	19	51	7	36 (27)	17	46	6	20 (24)	17	48	7	31 (22)
GH	18	13	36	6	-4 (13)	14	39	4	0 (17)	13	35	5	3 (14)	10	27	3	-3 (14)	11	31	5	2 (12)
VT	16	12	34	6	-2 (14)	16	44	4	17 (20)	18	50	7	16 (22)	14	39	5	9 (20)	12	34	5	11 (14)
SF	26	17	48	7	-3 (21)	19	53	5	19 (24)	19	52	7	14 (25)	14	38	5	1 (19)	17	46	7	19 (26)
RE	28	28	79	14	3 (31)	35	97	9	25 (43)	34	94	14	24 (47)	27	74	9	9 (38)	28	78	11	30 (43)
MH	24	12	33	5	-3 (14)	15	40	4	12 (17)	14	39	6	8 (18)	12	33	4	5 (17)	12	34	5	12 (17)

Note: ^¶ ^95%CI for population-based normative scores on SF-36 subscales [36].

* SEM (Standard error of measurement) = √within subjects variance; Derived from ANOVA model with 'time of follow-up' as the within subjects factor.

# MDC_ind _(Minimal detectable change at individual level) = 1.96*√2*SEM; MDC_grp _(Minimal detectable change at group level) = (1.96*√2*SEM)/√n.

PF = Physical Functioning, RP = Role Physical, BP = Bodily Pain, GH = General Health, VT = Vitality, SF = Social Functioning, RE = Role Emotional, MH = Mental Health.

#### Sensitivity: Individual changes

Sensitivity of SF-36 subscales to individual change was very low, as indicated by the high values of SEM and MDC_ind _(Table 5). The MDC_ind _in all study groups far exceeded the normative values of 95% CI (Table 5), indicating much greater amount of measurement error in SF-36 subscale in orthopedic settings than in the normative sample. Across all surgical groups, the GH subscale had the best sensitivity, with lowest values of MDC_ind _in all intervention groups. However a change as large as 27% or greater needed to occur on this subscale before it could be considered 'real'. RP and RE subscales were least sensitive to individual change with values of MDC_ind _ranging from 81 (ACL) to 91 (THR patients) and from 74 (APM) to 97 (THR patients), respectively.

#### Proportion improved or deteriorated

**Figure 3:**
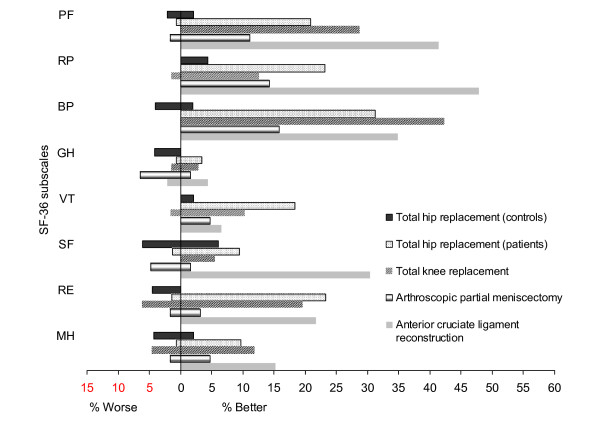
Proportion improved or deteriorated on SF-36 subscales across the study groups at first follow-up*. * Note: First follow-up was three months for APM and six months for TKR, THR, and ACL groups. PF = Physical Functioning, RP = Role Physical, BP = Bodily Pain, GH = General Health, VT = Vitality, SF = Social Functioning, RE = Role Emotional, MH = Mental Health.

The proportion of participants who could be classified as either improved or deteriorated during the follow-up is presented in Figure 3. Participants in the control group of the THR study were approximately equally likely to deteriorate or improve while in the intervention groups, the participants were more likely to improve. An exception was the GH subscale, with the vast majority classified as unchanged: 96% in THR (patients) and TKR groups, 93% in ACL group, and 92% in APM group. Overall, surgical group with the greatest proportion of patients who improved was ACL, followed by TKR and THR groups, with APM patients being generally least likely to improve.

### Population norm comparisons

**Figure 4:**
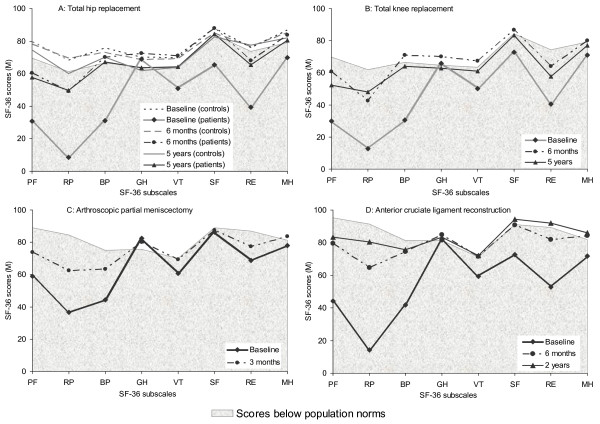
**Comparisons of SF-36 subscale scores of the study groups with population norms**. PF = Physical Functioning, RP = Role Physical, BP = Bodily Pain, GH = General Health, VT = Vitality, SF = Social Functioning, RE = Role Emotional, MH = Mental Health.

Figure 4 indicates that at baseline, all the surgical groups deviated most from the population norms on the RP subscale and were most similar to the norms on the GH subscale. As expected, the controls in the THR study changed little throughout the follow-up and were comparable to population norms at each assessment. At baseline, only GH scores were within the population norms for THR and TKR patients. The THR patients generally improved but were still below the population norms on PF, RP, and RE subscales at six months and five years follow-up (Figure 4a). TKR patients also generally improved, scoring slightly above the norm on the GH, BP, and VT subscales (Figure 4b), but below the norms on PF, RP, and RE at six months. At five years follow-up, TKR patients had a slight drop in their PF, BP, VT, and RE scores and were still below the norm on PF, RP, and RE subscales.

In the APM study, patients' baseline scores were slightly above the norm on the GH subscale and within the norm on SF and MH subscales. At three months follow-up, patients improved on PF, RP, BP, VT, and RE subscales but reached population norms on VT subscale only (Figure 4c). The ACL group had lower baseline scores than the norm on all subscales except GH. At six months, patients generally improved, but stayed below the norm on PF, RP, BP, and RE subscales. At two years follow-up, further improvements were recorded on RP and RE subscales, with patients scoring slightly above the norm on RE, but remaining below the norm on RP subscale (Figure 4d).

## Discussion

Orthopedic surgery is performed in response to a broad spectrum of conditions, including degenerative disorders and sports injury. We examined the magnitude and meaningfulness of changes in SF-36 subscales in four orthopedic populations and compared changes in patients' health status with the age and sex matched population norms. Large improvements (ES≥0.80) were observed on physical dimensions of the SF-36 (PF, RP, and BP subscales). Improvements on the mental and social dimensions (SF, RE, VT, and MH subscales) were small to moderate, while GH scores remained relatively unchanged during the study period. Group changes on all subscales but GH were clinically and statistically meaningful. Despite improvements, patients were still below the age and sex matched population norms on physical dimensions but scores on mental and social dimensions generally approached population norms following the surgery. On an individual level, floor and ceiling effects were observed on several subscales and the sensitivity to individual change was very low. Of the eight SF-36 subscales, the GH subscale had the best sensitivity to detect changes in health status of individual patients, although values of MDC_ind _were very high even on this subscale. PF subscale generally performed best, with no floor or ceiling effects and large changes in patients' scores following surgery however it had low sensitivity to change in individual or group scores.

Our results also indicate that overall, patients who underwent THR, TKR, APM, and ACL reconstruction surgery showed improvements in the health domains assessed by the SF-36 subscales. While the magnitude of the changes in SF-36 domains varied between the surgical groups, generally, greatest improvements were recorded for the physical dimensions, including physical function, role physical, and bodily pain, with more moderate changes in vitality, social functioning, role emotional, and mental health. Although no comparable data are currently available for APM, previous studies with THR, TKR, and ACL patients also documented greatest changes in the physical domains [18,38-42]. This study supports findings of past studies and extends them to a wider range of orthopedic surgery types.

Several researchers have previously recommended that interventions conducted with orthopedic populations should include at least one generic health status questionnaire in addition to condition-specific measures [8,41,43-45]. Disease-specific instruments, such as the Knee Injury and Osteoarthritis Outcome Score (KOOS), Western Ontario and McMaster Universities Osteoarthritis Index (WOMAC), and Arthritis Impact Measurement Scales (AIMS) for example, have been found reliable, valid, and sensitive measures of patient-reported outcomes in arthritis [20,46,47]. Disease-specific measures were also reported to be more sensitive in detecting change following surgical interventions than the generic instruments [8]. However, generic health status measures, such as SF-36, provide a broader insight into patients' quality of life and allow comparisons across conditions. Our results provide some support for the use of SF-36 to evaluate outcomes of THR, TKR, and ACL surgery, as improvements in vitality, social functioning, role emotional, and mental health of these surgical groups would have been missed if only disease-specific instruments were used.

In APM surgery, the changes in SF-36 scores were smaller than in other surgical groups. The mean age in the meniscectomy group was 45 years, implying a large proportion of degenerative meniscus tears in this group. Degenerative tear is a strong risk factor for future radiographic osteoarthritis and have been suggested to signal incipient knee OA [48]. Thus, the modest improvements seen in this group might be due to the surgery being performed for the wrong reason. A recent RCT in subjects with an MRI-verified meniscal tear compared meniscectomy and exercise with exercise alone and found no superior effect of meniscectomy, further questioning the effectiveness of meniscectomy in middle-aged people [49].

Another important finding in this study was that observed changes on all SF-36 subscales except GH were clinically and statistically meaningful at a group level. However, values of MDC_grp _in our study where higher than the established values of MCIC [36] for almost all subscales, indicating that at least some of the meaningful changes in group scores of orthopedic patients could not be detected with 95% confidence due to measurement error. Sensitivity of SF-36 subscales was even lower at an individual level, with very large changes in scores needed to occur before such changes could be classified as real with 95% confidence. The disparities in the amount of measurement error between ours and the normative samples [36] highlight the importance of evaluating outcome measures in the populations and settings for which these measures will be used. Poor sensitivity of SF-36 to individual change was previously observed in an analytical review of health status measures, with confidence intervals unacceptably wide to be of practical use for individual assessment [50] and in prospective follow-up of THR patients [17], raising concerns about the ability of SF-36 to reliably detect meaningful changes in health status of individuals. Information on sensitivity of a measure can potentially be used by clinicians and researchers to determine whether observed changes in the health status of individual patients or groups of patients reflect real changes as opposed to random variations. However, since our results suggest poor sensitivity of SF-36 subscales to individual change, we advise against using this questionnaire to monitor individual patients.

Previous studies with TKR, THR, and ACL patients reported that the GH subscale of SF-36 showed very little change in group scores after the surgery [17,39,40,42]. Similar findings were obtained in our study, with GH subscale showing little or no change across the study groups. However, group results are not necessarily a valid indicator of changes in health state of individuals, especially in situations where there are as many patients deteriorating as improving: when averaged for the whole group, the results may appear to suggest no change. Examination of individual scores in our study indicated that very few individuals could be classified as changed across the intervention groups on GH subscale. This finding extends the results of previous studies and underscores the importance of taking into account individual as well as group changes when evaluating outcomes in longitudinal studies [51].

Our results also indicate that patients in all intervention groups had general health scores comparable with the age and sex adjusted population norms. Lack of improvement in GH scores across the study groups is therefore not surprising as the participants were already in very good general health before the surgery. We also found that despite substantial improvements in health status over the study period, patients in the THR, TKR, APM, and ACL studies remained below the age and sex norms for the general population on several SF-36 subscales. While no data is currently available that compares outcomes of APM with the population norms, at least two previous investigations with THR and TKR patients [39,52] reported that patients who undergo these surgical interventions still fall short of age and sex adjusted population norms on health domains measured by SF-36.

We also found that floor and/or ceiling effects were present in most SF-36 subscales for nearly all intervention groups; hence the results of magnitude of changes (effect sizes) following orthopedic surgery need to be interpreted with caution, as changes can not be reliably estimated for individuals with extreme scores. The presence of floor and ceiling effects also indicates that SF-36 is not covering the full continuum of impairment and recovery in orthopedic populations. Substantial floor and ceiling effects for SF-36 scores were previously reported in other investigations [2,40], further indicating poor utility of SF-36 in orthopedics.

This study is subjected to some limitations. Firstly, it was not specifically designed to assess performance of SF-36 in different types of orthopedic surgery. Different methodologies were used and the study groups differed on some demographic variables. Therefore, some differences across groups may be related to study effects. Secondly, MCIC in the SF-36 domains are not well studied in orthopedic surgery, therefore we have used established population norms to gauge the amount of measurement error around individual change scores in orthopedic surgery settings. While the results indicate low sensitivity of SF-36 to individual change, future studies need to compare the MDC values with empirically derived estimates of MCIC following different types of orthopedic surgery. Finally, the presence of floor and ceiling effects on several SF-36 subscales suggests that the amount of change that could potentially occur for individual participants during the follow-up may have been influenced by their baseline scores, with greater possible range of change scores for individuals with midrange scores at baseline than for those who had more extreme baseline scores. As a result, within-subjects variability may have been underestimated, potentially distorting estimates of MDC [29,53].

One of the major strengths of this study is the use of data from four different types of orthopedic surgery. While several past studies investigated measurement properties of SF-36 in joint replacement surgery [7,9,17,38,45,54], to the best of our knowledge, ours is the first study to consider performance of SF-36 in THR, TKR, APM, and ACL reconstruction surgery simultaneously. Additional strengths of this study are the prospective design of the studies included and the high follow-up rates (65–100%). These aspects of study methodology serve to reduce bias and improve generilizability of results. Finally, we presented estimates of change in SF-36 subscale scores expressed in standardized units (ES) and in the original scale of measurement (MDC and SEM). While estimates of change in original scale of measurement have the advantage of being conceptually easy to interpret, ES can be used by clinicians and researchers to compare changes in patients' health status on different measures obtained in the same study, to evaluate efficacy of different interventions, or to compare results of different studies.

## Conclusion

Large to moderate meaningful changes in group scores were observed in all SF-36 subscales except GH across the intervention groups. Therefore, in orthopedic settings, the SF-36 can be used at a group level to show change in physical, mental, and social dimensions following different types of surgery and to make comparisons of the surgical groups with population norms. At an individual level however, SF-36 subscale had low sensitivity to individual change. Although further research is needed to establish the minimal clinically important change in SF-36 scores in orthopedic settings, we caution against using SF-36 to monitor health status of individual patients undergoing orthopedic surgery.

## Competing interests

The authors declare that they have no competing interests.

## Authors' contributions

LB participated in study design, performed the statistical analysis, and drafted the manuscript. RHO participated in study design and helped to draft the manuscript. AN carried out data collection (THR study). RB participated in study design and helped to draft the manuscript. EMR conceived of the study, participated in the design of the study, carried out data collection,TKR, APM, and ACL studies, and helped to draft the manuscript. All authors read and approved the final manuscript.
